# Supplementary material for: Labour-Market Characteristics and Self-Rated Health: Evidence from the China Health and Retirement Longitudinal Study
Source: Int J Environ Res Public Health. 2023 Mar 8;20(6):4748. doi: 10.3390/ijerph20064748 (PMC10048592; doi:10.3390/ijerph20064748)
Supplement: Supplementary file 1 [file ijerph-20-04748-s001.zip › ijerph-2161438-supplementary.pdf]

Supplementary

Table S1. Distribution of self-rated health measured by 5-point scale among the working population.

Self-rated health	N (%)
**Very good**	396 (10.25)
**Good**	919 (23.78)
**Fair**	1,998 (51.71)
**Poor**	490 (12.68)
**Very poor**	61 (1.58)
Total	3,864

Table S2. Association between each labour market characteristic and poor self-rated health in working population according to region.

	East N=1613 (42%)	Central N=1056 (27%)	West N=903 (23%)	Northeast N=292 (8%)	Overall effect of interactions
	OR (95% CI) Age- and sex-adjusted	OR (95% CI) Age- and sex-adjusted	OR (95% CI) Age- and sex-adjusted	OR (95% CI) Age- and sex-adjusted	Age- and sex-adjusted
**Employment status**					
Employed	Ref	Ref	Ref	Ref	Chi^2=4.89
Self-employed	1.24 (0.86,1.79)	1.16 (0.79,1.70)	1.09 (0.72,1.65)	1.55 (0.75,3.22)	P=0.77
Unpaid family business	2.84 (1.72,4.69)[**]	2.05 (1.11,3.81)[*]	2.13 (1.22,3.71)[*]	0.95 (0.24,3.73)	
Not enough information to classify	2.70 (1.17,6.23)[*]	1.89 (0.75,4.75)	3.91 (1.99,7.68)[**]	-	
**Weekly working hours**					
1-39 h/week	2.63 (1.52,4.53)[*]	1.08 (0.63,1.85)	1.48 (0.85,2.59)	9.78 (2.02,47.28)[*]	Chi^2=22.16
40-49 h/week	Ref	Ref	Ref	Ref	P=0.04
50-59 h/week	0.99 (0.54,1.83)	0.96 (0.54,1.70)	0.93 (0.49,1.76)	4.42 (0.84,23.25)	
≥60 h/week	1.63 (0.96,2.76)	0.88 (0.53,1.47)	1.52 (0.90,2.58)	5.76 (1.26,26.27)[*]	
Missing[a]	3.84 (2.18,6.77)[**]	0.88 (0.47,1.67)	2.33 (1.30,4.15)[*]	8.97 (1.60,50.31)[*]	
**Public or private sectors**					

Public	Ref	Ref	Ref	Ref	Chi^2=3.12
Private	2.13 (1.25,3.64)*	1.53 (0.97,2.42)	1.35 (0.88,2.07)	1.12 (0.56,2.24)	P=0.37
Missing[b]	-	-	11.14(0.95,129.93)	-	
**Earned income**					
1 (Richest)	Ref	Ref	Ref	Ref	Chi^2=16.89
2	1.32 (0.70,2.46)	1.69 (0.80,3.57)	1.56 (0.68,3.56)	4.89 (0.52,45.88)	P=0.33
3	1.08 (0.57,2.06)	1.01 (0.45,2.25)	1.74 (0.75,4.02)	2.88 (0.25,33.32)	
4	1.71 (0.91,3.22)	1.80 (0.89,3.63)	1.75 (0.79,3.88)	14.57(1.79,118.62)*	
5 (Poorest)	3.34 (1.86,6.01)**	2.35 (1.15,4.76)*	2.66 (1.24,5.71)*	13.63(1.65,112.81)*	
Missing[b]	3.48 (1.97,6.16)**	1.78 (0.87,3.64)	4.23 (2.03,8.78)**	9.57 (1.14,80.56)*	
**Occupation**					
Managers and professionals	Ref	Ref	Ref	Ref	Chi^2=11.23
Technicians and associate professionals	0.20 (0.03,1.46)	1.19 (0.43,3.31)	0.67 (0.19,2.35)	-	P=0.42
Clerks and workers	0.99 (0.66,1.50)	1.29 (0.84,1.98)	1.04 (0.67,1.63)	0.67 (0.30,1.50)	
Elementary occupations	0.94 (0.52,1.70)	1.01 (0.54,1.92)	1.19 (0.64,2.19)	1.87 (0.59,5.94)	
Missing[a]	2.50 (1.47,4.25)*	1.82 (1.00,3.33)	1.82 (1.07,3.10)*	0.85 (0.25,2.95)	
**Years working on current job**					
≤10 years	Ref	Ref	Ref	Ref	Chi^2=6.87

>10 years	0.87 (0.61,1.24)	1.19 (0.82,1.74)	0.85 (0.58,1.25)	0.39 (0.18,0.86)*	P=0.33
Missing[b]	2.02 (1.30,3.12)*	1.98 (1.17,3.35)*	1.54 (0.95,2.50)	1.48 (0.46,4.74)	
**Employer-provided/ self-employed covered insurances**					
At least one insurance	Ref	Ref	Ref	Ref	Chi^2=9.73
None	1.12 (0.74,1.69)	1.10 (0.69,1.77)	1.68 (1.06,2.66)*	3.29 (1.28,8.42)*	P=0.14
Missing[b]	1.55 (0.88,2.72)	1.22 (0.64,2.31)	1.88 (1.04,3.40)*	1.07 (0.29,3.98)	

Abbreviations: *OR* odds ratio, *CI* confidence interval.

[a]Implausible value or not answered.

[b]Implausible values, not answered or not asked.

*p<0.05, **p<0.001

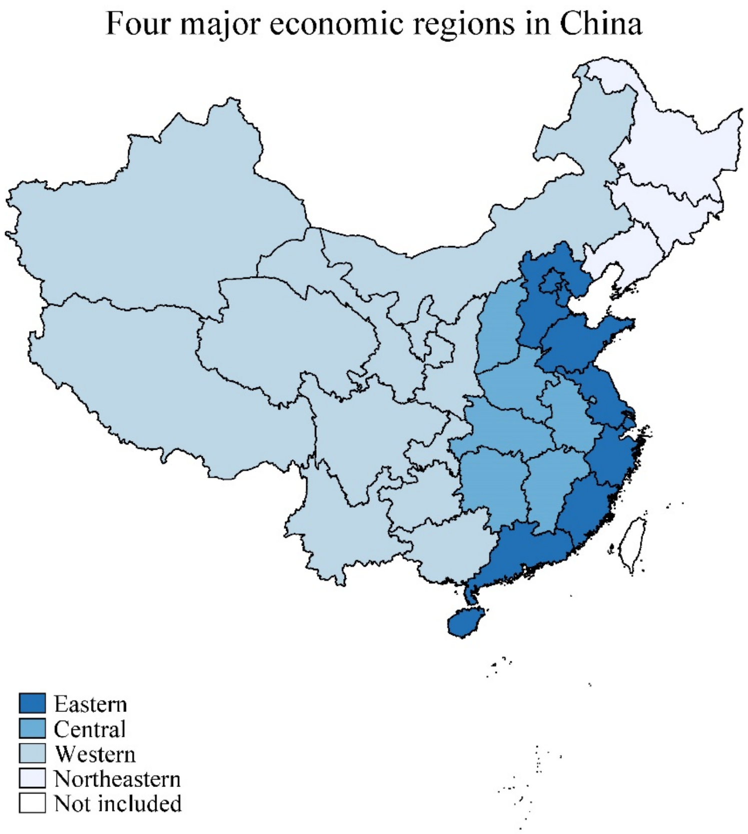

Figure S1. Four major economic regions in China.
